# Response to immunotherapy in a patient with anaplastic thyroid cancer: A case report

**DOI:** 10.1097/MD.0000000000026138

**Published:** 2021-08-13

**Authors:** Luming Zheng, Ling Li, Qingqing He, Meng Wang, Yunhan Ma, Jian Zhu, Yanchen Li, Xiaokang Fu, Yaxuan Zhang

**Affiliations:** a Department of General Surgery, 960th Hospital of the People's Liberation Army, Jinan, Shandong, P. R. China; b Yinfeng Gene Technology Co Ltd, CORA, Jinan, Shandong, P. R. China.

**Keywords:** anaplastic thyroid carcinoma, camrelizumab, immunotherapy, PD-1 inhibition

## Abstract

**Rationale:**

Anaplastic thyroid carcinoma (ATC) is an aggressive malignancy that is almost always fatal and lacks effective systemic treatment options. Current treatments of ATC include surgery, radiation, and chemotherapy, used in combination when possible. In the aspect of immunotherapy, the biomarker of TMB-H and MSI-H may suggest that patients benefit from pembrolizumab. Programmed cell death-ligand 1 (PD-L1) is highly expressed in ATC but has not been written into the guidelines or approved by the FDA as a biomarker for thyroid cancer immunotherapy.

**Patient concerns:**

A 55-year-old woman was admitted to our hospital because of a slight right-sided neck enlargement in November 2019.

**Diagnoses:**

The clinical diagnosis was ATC, pT3bN0M0, and stage IVB.

**Interventions:**

Oral administration of apatinib (250 mg 3 times daily) was initiated after surgery, but some unpleasant side effects emerged after 1 month of treatment. Next-generation sequencing revealed that the tumor harbored 2 mutations, HRAS p.Q61R and TP53 p.P278S, and PD-L1 staining was positive with a high expression. Thus, camrelizumab (programmed cell death protein 1 inhibitor) was combined with apatinib, and apatinib was changed to 250 mg once a day from March 2020.

**Outcomes:**

No adverse reactions were observed after the treatment immunotherapy combined with antiangiogenic drugs. Currently, the survival time of patients is more than 11 months, and the quality of life is not affected.

**Conclusion:**

This case suggests that immunotherapy in patients with ATC based upon PD-L1 evaluation provides a therapeutic option. Targeting programmed cell death protein 1/PD-L1 may provide a much-needed treatment option for patients with advanced ATC.

## 1 Introduction

Anaplastic thyroid carcinoma (ATC) is a highly aggressive malignant thyroid tumor representing 1% to 2% of all thyroid cancers. The prognosis is poor, with a mortality rate of >90% and a mean survival of 6 months after diagnosis.^[1]^ Once diagnosed, the clinical stage is 4 defined by US NCCN.^[2]^ Current treatments of ATC include surgery, radiation, and chemotherapy, used in combination when possible.^[3,4]^ However, the overall response rates are typically short, and the various therapies probably do not affect overall survival at the population level.^[5]^ Given the poor prognosis and paucity of current therapies, molecular profiling of tumors may provide additional opportunities for therapeutic targets. However, a critical unmet need remains for effective therapy for patients with BRAF wild-type, RET wild-type, and NTRK wild-type disease.^[6,7]^ In the aspect of immunotherapy, the biomarker of TMB-H and MSI-H may suggest that patients benefit from pembrolizumab.^[8]^ Programmed cell death-ligand 1 (PD-L1) is highly expressed in ATC but has not been written into the guidelines or approved by the FDA as the biomarker for thyroid cancer immunotherapy.^[9]^ Here, we describe a patient who benefited from programmed cell death 1 (PD-1) inhibitor camrelizumab combined with antiangiogenic targeting drugs apatinib after operation.

## 2 Case presentation

**Figure 1:**
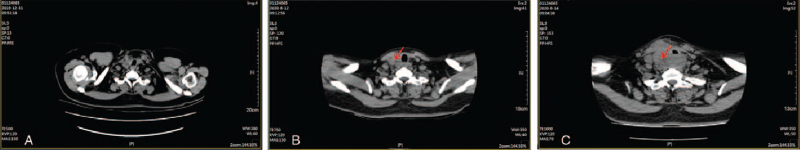
Axial view of CT scans of the neck showing the primary lesion and metastatic lymph. (A) Before camrelizumab treatment (December 31, 2019). (B) Six months after camrelizumab treatment (June 12, 2020). (C) Eight months after camrelizumab treatment (August 14, 2020). CT = computed tomography.

A 55-year-old woman was referred to our hospital because of a slight right-sided neck enlargement in November 2019. Ultrasound revealed that the nodular degeneration of the right lobe of the thyroid was accompanied by annular calcification, with unclear boundary, mixed and uneven internal echo, and enlargement of the bilateral lymph node. The patient refused fine-needle aspiration cytology under ultrasound guidance. Subsequently, total thyroidectomy and central and right lateral cervical lymph node dissection were performed on November 27, 2019. The clinical diagnosis was ATC, pT3bN0M0, and stage IVB. The patient refused systemic radiation and chemotherapy due to personal reasons. Considering her wishes, oral administration of apatinib (250 mg 3 times daily) was initiated after surgery, and axial view of computed tomography (CT) scans of the neck showed that the primary lesion was stable (Fig. 1A). However, some adverse events of grade 3 emerged after 1 month of treatment, including gingival redness, swelling, and bleeding. As a result of these undesirable effects, after obtaining consent from the patient, the surgically resected tissues were used to test 600 tumor-related genes for targeted therapy or immunotherapy. Next-generation sequencing (NGS) revealed that the tumor harbored 2 mutations, HRAS p.Q61R and TP53 p.P278S, and PD-L1 staining was positive with a high expression (35% of tumor cells). These findings suggested that the patient might benefit from immunotherapy. Camrelizumab (AiRuiKa) is a PD-1 inhibitor developed by Jiangsu Hengrui Medicine Co. Ltd. for the treatment of various malignancies.^[10]^ It was combined with apatinib, and apatinib was changed to 250 mg once a day from March 2020. The medication was well tolerated, with no adverse events, and CT revealed slight local recurrence in June (Fig. 1B). The patient was admitted to the hospital on August 14, 2020, and CT revealed local recurrence (Fig. 1C). Upon consent from the patient, local radiotherapy was added to the treatment regimen. The patient continues to be in the clinical stable stage 11 months after beginning treatment with camrelizumab and apatinib.

## 3 Discussion

In all thyroid cancer types, the management of ATC remains most challenging. Previous analyses have reported that the extent of traditional therapeutics, such as surgery, radiotherapy, and chemotherapy, may be associated with the length of survival.^[11,12]^ In most cases, however, the diagnosis of ATC is in the advanced stage. Patients with ATC are considered inoperable and show an adverse reaction rate to systemic chemotherapy, so the overall treatment effect is very poor. Our patient was not amenable to the above conventional treatment options considering her personal reasons after surgery. New therapeutic strategies were urgently needed.

Given that increased VEGFR expression has been found in the microvascular endothelial cells of ATC tumor specimens,^[13]^ agents targeting VEGFR can block the effects of vascular endothelial growth factors and play antiangiogenic and antitumor roles in solid tumors. Apatinib, a novel tyrosine kinase inhibitor that has highly selective competition in the ATP binding site of VEGFR-2, blocks down pathways and inhibits tumor angiogenesis.^[14,15]^ Moreover, overwhelming efficacy has been achieved in radioiodine-refractory differentiated thyroid cancer.^[16]^ All the above evidence favored apatinib as an ideal choice for our patient. As a result, gratifying outcomes were achieved regarding a durable response, but she reduced apatinib dosage because of some unpleasant side effects.

The era of incurable ATC is gradually being replaced by molecular-based personalized therapies, which integrate multidisciplinary therapies including surgery, immunotherapy, radiation therapy, and targeted therapy.^[17]^ In 2018, the FDA approved dabrafenib in combination with trametinib for the adjuvant treatment of patients with BRAF V600 mutation-positive ATC who could not be treated by resection or whose cancer cells had spread to other parts of the body. This therapy has greatly improved the survival of such patients. Additionally, the value of gene examinations needs to be thoroughly examined in decision-making using an appropriate sample size of patients with ATC because of their success in methodology.^[18]^ The above evidence demonstrated that the patients underwent NGS molecular detection for targeted therapy or immunotherapy, and NGS revealed that PD-L1 staining was positive with a high expression.

Currently, PD-1 blockades are widely used in multiple tumor types, and effective responses have been obtained. In recent years, the use of immunotherapy has increased significantly in the current ATC treatment research, although the improvements in survival cannot be directly attributed to immunotherapy alone due to a variety of confounding factors, especially the use of synchronous targeted therapy. Even though single-agent immunotherapy has not shown broad prospects in the treatment of ATC,^[19]^ the potential of combining targeted therapies with immunotherapy agents such as pembrolizumab^[20]^ and atezolizumab^[21]^ is being studied in completed and/or ongoing clinical trials, with significant results to date. In addition, a clinical trial reported that spartalizumab promises clinical activity and a good safety profile in a patient population with an aggressive incurable disease and short life expectancy targeting PD-L1-positive advanced ATC.^[22]^ In our case, overexpression of PD-L1 may be a potential reason for the patient to benefit from immunotherapy.

Given the poor prognosis of ATC, patients with ATC should discuss care goals with a multidisciplinary team. Multimodal therapy is associated with longer overall survival in patients with ATC. In the past few years, the treatment of ATC has evolved from palliative care and hospice care to effective highly specialized molecular-based personalized therapies and surgery when appropriate, regardless of the disease stage. Immunotherapy may delay and/or prevent the emergence of drug-resistant mutations, and it can maintain targeted therapies so that disease control can be sustained. This case suggests that immunotherapy in patients with ATC based upon PD-L1 evaluation provides a therapeutic option. Targeting PD-1/PD-L1 may provide a much-needed treatment option for patients with advanced ATC. Additional investigations will be necessary to characterize the mechanism for the efficacy observed with immune checkpoint inhibition. Further research on immunotherapy will be carried out in ATC in the future, and the practicability and efficacy of the treatment of these patients need to be further studied.

## Acknowledgments

We would like to thank Yinfeng Gene Technology Co., Ltd. for the help in organizing the data and revising this article.

## Author contributions

**Conceptualization:** Luming Zheng.

**Data curation:** Qingqing He, Ling Li.

**Investigation:** Meng Wang, Yunhan Ma.

**Supervision:** Yunhan Ma.

**Validation:** Jian Zhu, Yanchen Li, Xiaokang Fu.

**Writing – original draft:** Ling Li, Qingqing He.

**Writing – review & editing:** Yaxuan Zhang.
